# A Rare Case of Streptococcus equi Infection: A Report and Brief Review of the Literature

**DOI:** 10.7759/cureus.99040

**Published:** 2025-12-12

**Authors:** Bhavik Singh, Zoheb I Sulaiman, Eileen M Raynor

**Affiliations:** 1 Department of Clinical Education, Doctor of Osteopathic Medicine Program, Philadelphia College of Osteopathic Medicine, Suwannee, USA; 2 Department of Infectious Diseases, Piedmont Atlanta Hospital, Atlanta, USA; 3 Department of Head and Neck Surgery and Communication Sciences, Duke University Medical Center, Durham, USA

**Keywords:** bacteremia, equine exposure, prosthetic joint infection, septic arthritis, streptococcus equi subspecies zooepidemicus, zoonotic infection

## Abstract

We present a rare case of *Streptococcus (S.) equi* subspecies zooepidemicus (SEZ) bacteremia in a 73-year-old male with a history of Parkinson’s disease, pulmonary embolism, and recent left knee total arthroplasty who developed fever and sepsis complicated by septic arthritis of a prosthetic knee and deep vein thrombosis (DVT). Blood cultures grew *S. equi* in four out of four bottles, and history revealed frequent horse exposure, suggesting zoonotic transmission with hematogenous seeding of the prosthetic joint. The diagnosis was further challenged by systemic computed tomography (CT) abnormalities and the concurrent DVT, which initially obscured the infectious source. The patient was treated with three days of piperacillin-tazobactam before de-escalation to intravenous ceftriaxone for six weeks, followed by 6 months of oral amoxicillin-clavulanate, which was then extended for an additional 12 months based on clinical response. SEZ causing prosthetic joint infection is exceptionally rare, and this case underscores the importance of recognizing zoonotic pathogens as potential causes of hematogenous prosthetic joint infections, particularly in immunocompromised hosts.

## Introduction

*Streptococcus (S.) equi* subspecies zooepidemicus (SEZ) is a beta-hemolytic group C streptococcus commonly found in horses [1]. It is the cause of “Strangles”, which is a highly infectious upper respiratory tract disease followed by abrupt pyrexia, pharyngitis, yellow to clear discharge, and abscess formation in horses [2]. Intramuscular vaccinations for horses to combat Strangles, such as Strangvac, demonstrate a 94% efficacy after the third vaccination [3]. Though primarily an equine pathogen, zoonotic infections have been reported in humans, often linked to raw dairy consumption or direct animal contact [4,5].

Earlier literature described a small number of human cases; however, more recent reports suggest that the true incidence is likely underrecognized because contemporary epidemiologic data remain limited and sporadically reported [6]. Human infections can manifest as bacteremia, endocarditis, meningitis, and septic arthritis, particularly in immunocompromised individuals.

SEZ involvement in prosthetic joint infections remains exceptionally rare, and multisystem involvement, such as concurrent thrombotic or systemic inflammatory complications, is even less frequently described. This case highlights a unique presentation of SEZ bacteremia complicated by septic arthritis of a prosthetic knee and deep vein thrombosis in the setting of significant equine exposure, adding to the limited literature on zoonotic musculoskeletal infections.

## Case presentation

A 73-year-old male with a history of Parkinson’s disease, hypertension, hyperlipidemia, pulmonary embolism, and left total knee arthroplasty in March 2022 presented in June 2023 with 4 days of fever, shaking chills, and progressive weakness. His temperature was 103.3 °F, his systolic blood pressure was in the 80s, and laboratory results showed leukocytosis (WBC 19.5 x103/μL; normal range 4,000-11,000 µL) with neutrophil predominance and bandemia. Inflammatory markers were noted to be elevated on admission. Blood cultures revealed gram-positive cocci in chains, which were negative for *Staphylococcus aureus*, *Streptococcus pyogenes*, *Streptococcus agalactiae*, and Viridans group *Streptococci*. Further identification using matrix-assisted laser desorption ionization-time of flight (MALDI-TOF) mass spectrometry confirmed *S. equi* subspecies zooepidemicus in four out of four bottles.

The patient endorsed frequent contact with multiple horses, suggesting potential zoonotic transmission. He also reported right arm trauma two weeks prior while handling a horse, though no wound breakdown or signs of infection were documented; therefore, this could not be confirmed as a portal of entry. In the week leading up to admission, he noticed increasing discomfort, swelling, and warmth of his left knee, which had previously undergone arthroplasty. These symptoms progressed alongside his systemic decline and raised clinical concern for septic arthritis.

**Figure 1:**
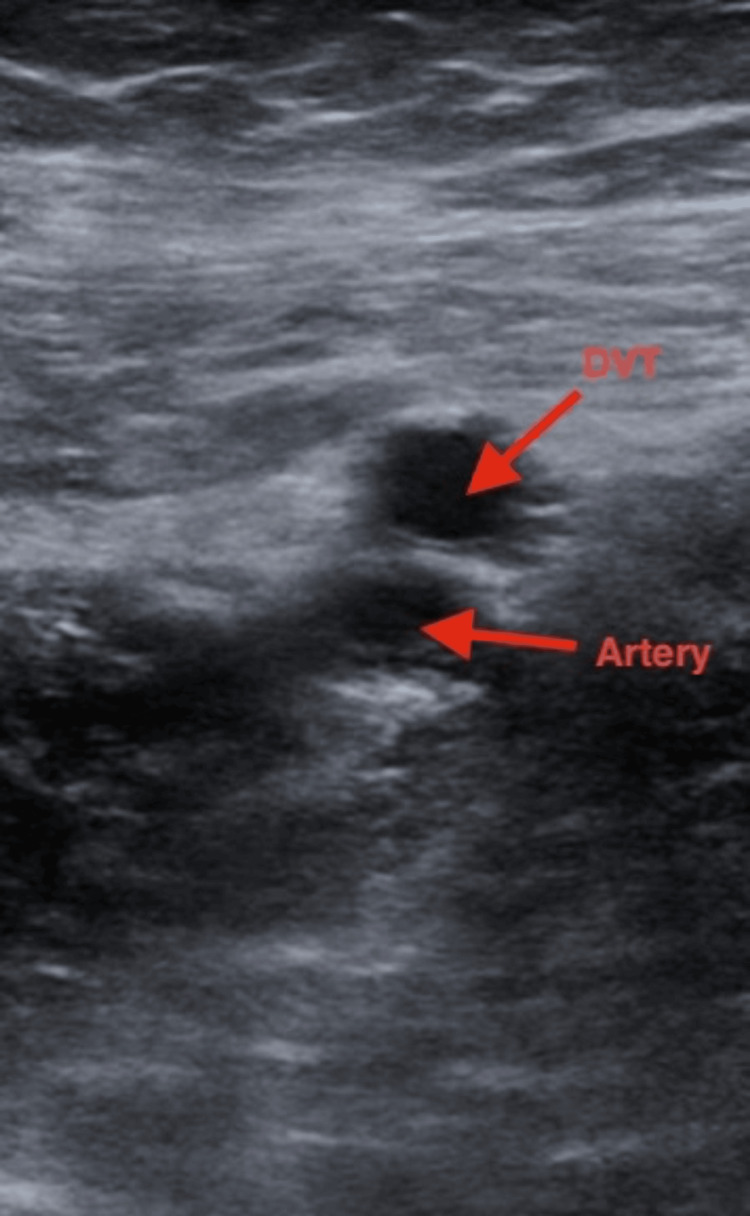
Ultrasound demonstrating right lower extremity deep vein thrombosis Transverse view demonstrating a noncompressible femoral vein containing intraluminal echogenic material (upper arrow), consistent with thrombosis. The adjacent femoral artery (lower arrow) remains patent.

**Figure 2:**
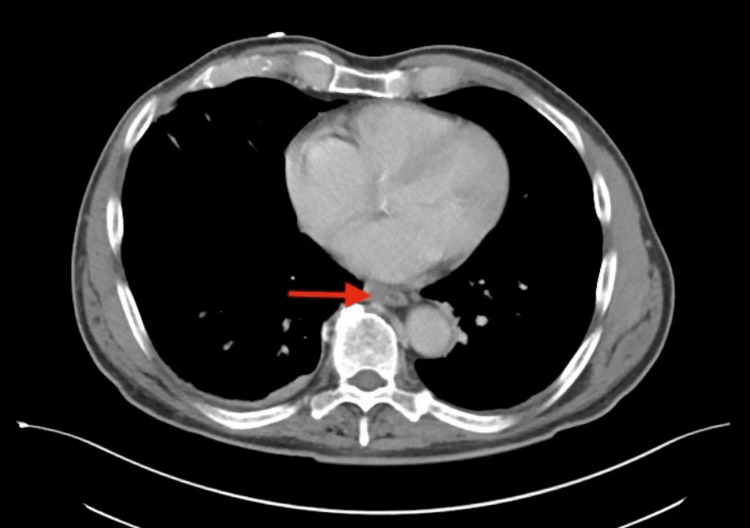
Axial contrast-enhanced computed tomography of the chest The image demonstrates circumferential thickening of the mid-to-distal esophagus (arrow) without focal mass or obstruction. The significance of this finding is uncertain and may represent a nonspecific inflammatory response.

**Figure 3:**
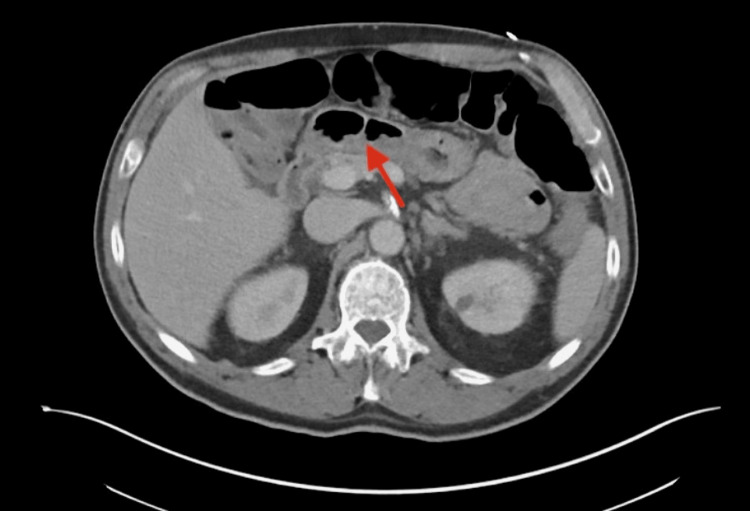
Axial contrast-enhanced computed tomography of the abdomen and pelvis Axial contrast-enhanced CT image of the pelvis demonstrates circumferential thickening of the rectal wall (arrow) without discrete abscess formation or mass lesion. The findings are consistent with diffuse inflammatory or infectious changes, though the significance of this finding is uncertain and may represent a nonspecific inflammatory response.

Imaging obtained during early hospitalization revealed an acute right lower-extremity deep vein thrombosis on Doppler ultrasound (Figure 1). While DVT may occur in the setting of systemic inflammation or immobility, a direct causal relationship with SEZ infection could not be determined. CT chest, abdomen, and pelvis demonstrated diffuse esophageal thickening (Figure 2) and rectal wall thickening (Figure 3). As the patient denied dysphagia, abdominal pain, or rectal bleeding, these findings were interpreted with caution and may have reflected nonspecific inflammatory changes rather than direct gastrointestinal involvement.

During admission, he developed persistent atrial flutter requiring adenosine and intravenous metoprolol boluses. His left knee was warm, erythematous, and tender on examination. Arthrocentesis performed two days later revealed purulent synovial fluid containing 48,520 WBCs (normal range <200 cells/µL) with 72% neutrophils (normal <25%), consistent with a septic arthritis profile. No crystals were identified. Synovial cultures obtained after washout did not yield additional organisms; however, susceptibility testing from the bloodstream isolate demonstrated β-lactam susceptibility, including to ampicillin, cefotaxime, and ceftriaxone, guiding subsequent antibiotic management. During his hospital course, he developed persistent atrial flutter that required treatment with adenosine and IV metoprolol.

Given the patient’s presentation with sepsis of unclear source, he was empirically started on intravenous piperacillin-tazobactam for broad-spectrum coverage. After three days, blood culture identified *S.* *equi *subspecies zooepidemicus, and susceptibilities supported β-lactam therapy. Treatment was subsequently narrowed to intravenous ceftriaxone following infectious disease and orthopedic consultation. Surgical washout of the infected prosthetic knee joint was performed three days later, with retention of the prosthetic hardware.

Follow-up CT imaging of the chest, abdomen, and pelvis performed after initial stabilization demonstrated interval resolution of the previously noted esophageal, gastrointestinal, and rectal wall thickening. These imaging improvements occurred prior to transition to oral suppressive therapy, correlating with early clinical response to intravenous antibiotics.

Following washout, the patient continued intravenous ceftriaxone 2 grams every 24 hours for six weeks, completed in mid-July 2023, in accordance with prosthetic joint infection guidelines for retained hardware. He was then transitioned to oral suppressive therapy with amoxicillin-clavulanate 875 mg every 12 hours for 6 months, based on the virulence of SEZ, the hematogenous source, and the decision to retain the prosthesis. After shared decision-making with infectious disease specialists, suppressive therapy was extended for an additional 12 months to reduce the risk of relapse, given the organism's ability to invade deep tissues and the presence of retained hardware. His planned suppressive antibiotic course was scheduled through December 2024.

## Discussion

Our findings align with previous reports that SEZ is an opportunistic zoonotic pathogen with the potential to cause severe invasive infections in humans, most often following direct or indirect exposure to horses or consumption of contaminated animal products. Reported human infections include bacteremia, endocarditis, meningitis, and septic arthritis, particularly in immunocompromised individuals [5]. Of note, prosthetic joint infection due to SEZ remains exceedingly rare, with only isolated cases documented worldwide. This case contributes additional insight by demonstrating hematogenous seeding of a prosthetic joint accompanied by multisystem findings, including deep vein thrombosis and gastrointestinal wall abnormalities.

Diagnostic considerations and organism identification

SEZ shares phenotypic characteristics with other Group C *streptococci*, which can lead to misidentification without confirmatory testing. In this case, initial organism characterization excluded *Staphylococcus aureus, Streptococcus pyogenes, Streptococcus agalactiae*, and Viridans group *Streptococci*, and definitive identification was achieved using MALDI-TOF mass spectrometry. This highlights the importance of advanced diagnostic platforms when unusual streptococcal species are suspected. Bloodstream susceptibility testing demonstrated β-lactam susceptibility, which guided therapeutic choice.

Comparison with previously reported human SEZ cases

Several case clusters, including a well-characterized series from Jeju Island, South Korea, had documented multiple infections caused by *S.* *equi *subsp. zooepidemicus, including bacteremia, pyogenic spondylitis, and necrotizing myositis [4]. While invasive, none reported prosthetic joint involvement. Compared with these cases, our patient’s presentation is distinguished by hematogenous infection of an orthopedic implant and concurrent DVT, as well as nonspecific gastrointestinal thickening on imaging. These findings expand the recognized clinical spectrum of SEZ infection, particularly in individuals with retained prosthetic material.

Orthopedic management and rationale for antibiotic strategy

The patient underwent surgical washout of the prosthetic knee with retention of the hardware, a management approach commonly used when components remain stable and infection is identified early. Retention, however, increases risk for persistent infection or relapse, necessitating prolonged antimicrobial therapy. Intravenous ceftriaxone was selected based on β-lactam activity, established efficacy against SEZ, and a favorable dosing profile compared with alternatives such as penicillin.

After completing six weeks of intravenous therapy, the patient was transitioned to suppressive oral amoxicillin-clavulanate. The decision to extend suppressive therapy for a total of 18 months was made collaboratively with infectious disease specialists and reflects several factors: the virulence of SEZ, hematogenous origin of infection, presence of retained hardware, and the paucity of data guiding management of prosthetic infections due to this organism [7]. Long-term suppression is consistent with prosthetic joint infection guidelines for cases in which hardware cannot be removed and the causative organism is susceptible to safe, well-tolerated oral agents.

Ancillary findings: DVT and gastrointestinal wall thickening

The acute right femoral deep vein thrombosis identified on ultrasound occurred in the setting of systemic inflammation and reduced mobility. Although temporally associated, a direct pathophysiologic link between SEZ infection and thrombosis cannot be established. Similarly, the esophageal and rectal wall thickening seen on CT imaging was nonspecific. These findings were resolved on follow-up imaging after initiation of intravenous therapy, but gastrointestinal involvement is not a recognized feature of SEZ infection, and its significance remains uncertain.

Limitations

Genomic comparison of our isolate to previously documented strains was not available; thus, broader conclusions regarding geographic or phylogenetic relationships cannot be made. Further research is needed to characterize the spectrum of virulence, tissue tropism, and optimal management strategies for SEZ infections involving prosthetic material.

## Conclusions

This case demonstrates that SEZ can cause severe human infection and underscores the importance of obtaining a careful animal exposure history in septic patients, particularly those with prosthetic devices. Even minor or remote equine contact may precede hematogenous seeding of a prosthetic joint, emphasizing the need for early imaging, joint evaluation, and multidisciplinary management with infectious disease and orthopedic specialists. Because the hardware was retained, prolonged suppressive antibiotic therapy was necessary to reduce the risk of relapse. Compared with prior reports, this case expands current understanding by illustrating prosthetic joint involvement and multisystem findings following equine exposure. Continued research is needed to clarify optimal management for SEZ infections involving orthopedic implants.
